# Operant Assessment of DMTP Spatial Working Memory in Mice

**DOI:** 10.3389/fnbeh.2019.00193

**Published:** 2019-08-27

**Authors:** Jasper Teutsch, Dennis Kätzel

**Affiliations:** Institute of Applied Physiology, Ulm University, Ulm, Germany

**Keywords:** spatial working memory, delayed-matching-to-position, guanfacine, modafinil, combined attention and memory (CAM) task

## Abstract

Working memory (WM) is required to bridge the time between the moment of sensory perception and the usage of the acquired information for subsequent actions. Its frequent and pharmacoresistent impairment in mental health disorders urges the development of rodent paradigms through back-translation of human WM tests, ideally avoiding the confounds of alternation-based assays. Here we show, that mice can acquire a delayed-matching-to-position (DMTP) operant spatial WM (SWM) paradigm that is akin to the *combined attention and memory* (CAM) task previously developed for rats, and that relies on a 5-choice wall [5-CSWM, 5-choice based operant testing of SWM (5-CSWM)]. Requiring ca. 3 months of daily training with a non-illuminated operant box in the default state, mice could attain a performance level of ≥70% choice accuracy with short (2 s) delays in the DMTP 5-CSWM task. Performance decreased with extended delays, as expected for WM processes. Modafinil (15 and 30 mg/kg) and guanfacine (0.3 and 1 mg/kg) showed no consistent efficacy in enhancing task performance. We also found, that mice did not improve beyond chance level, when trained in the DNMTP-version of the 5-CSWM. Our results outline the methodical possibility and constraints of assessing spatial WM in mice with an operant paradigm that provides high control over potentially confounding variables, such as cue-directed attention, motivation or mediating strategies like body-positioning.

## Introduction

Working memory (WM) in humans is the capacity to actively maintain and manipulate recently acquired sensory information at the forefront of conscious attention (Baddeley and Hitch, 1974; Baddeley, 1992). It is impaired in some neurological and the majority of psychiatric disorders (Millan et al., 2012), including schizophrenia (Barch and Smith, 2008). Pathologically occurring WM deficits are often pharmacoresistant; e.g., guanfacine was ineffective in patients with schizophrenia (Friedman et al., 2001) as well as in a delayed-matching-to-position (DMTP) paradigm in healthy humans (Jäkälä et al., 1999b), and modafinil was found effective in schizophrenia only in a subset of studies (Scoriels et al., 2013). Therefore, translational rodent assays, which can predict WM-enhancing effects in humans are sought-after (Barch et al., 2009).

**Figure 1:**
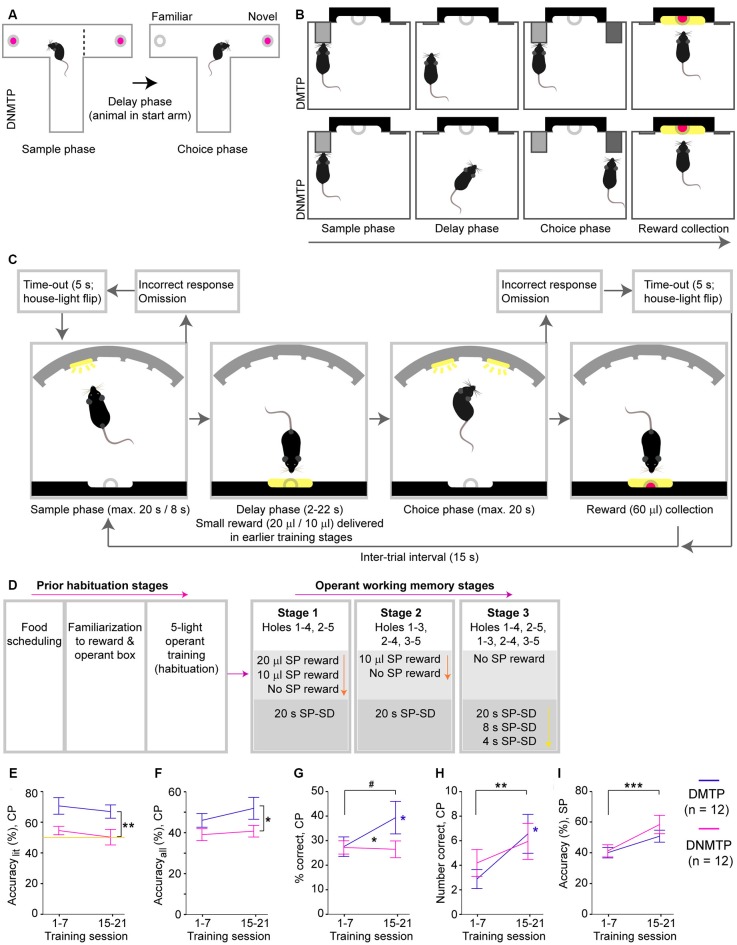
Sequence, training schedule and acquisition of a 5-choice-based operant working memory (WM) task in mice. **(A)** Scheme of the T-maze rewarded alternation spatial WM (SWM) task, which follows a delayed-non-matching-to-position (DNMTP, win-shift) paradigm, whereby the goal arm visited in the sample phase (SP, left) has to be avoided in the choice phase (CP, right) to obtain a reward (pink). However, mice also intrinsically prefer the correct goal arm as it is more novel than the incorrect goal arm. **(B)** Simple lever-based operant testing of SWM can follow either a delayed-matching-to-position (DMTP, top) or a DNMTP (bottom) paradigm, whereby a previously presented lever (gray, SP) has to either be pressed again (DMTP) or avoided in favor of another lever (DNMTP, dark gray) in the CP to obtain a reward. In both paradigms, mice can encode the future correct choice by placing their body in front of the correct lever during the delay phase. **(C)** 5-choice based operant testing of SWM (5-CSWM) reduces this confound of the lever-based task and offers more options to manipulate task difficulty: mice need to poke into an illuminated hole in the SP, return to the opposite wall during the delay phase, and then poke the previously illuminated (DMTP) or another illuminated (DNMTP) hole in the CP to obtain a large reward (pink). Omissions or pokes into incorrect holes during the SP or CP are punished by abortion of the ongoing trial, resulting lack of reward and a 5-s timeout during which the state of the house-light is flipped (switched off, if animals are otherwise trained with illuminated house-light, *light* protocol; switched on, if animals are otherwise trained without illuminated house-light, *dark* protocol). **(D)** Training schedule for acquisition of the task, with colored arrows representing transitions from one stage to the next over time, depending on performance (see Supplementary Methods). Prior habituation stages (left) include food restriction, delivery of the milk reward in the operant box and the acquisition of the basic operant cycle in which mice need to poke any hole of the 5-choice wall (all of which are illuminated) to obtain a reward. Subsequently, SWM training starts (right), whereby the number of options of co-illuminated holes in the CP increases across the main stages (pink arrow), and the amount of milk reward (orange arrow) or, later, the stimulus duration (SD) in the SP (yellow arrow) decreases. **(E–I)** Performance of mice trained in the DMTP (purple) or the DNMTP (pink) paradigm averaged within the first (left) and the third (right) block of seven training sessions for WM performance—accuracy_lit_ **(E)**, accuracy_all_ **(F)**, and percent correct responses **(G)**, number of correct responses made in the CP **(H)**, and the attentional accuracy with which an illuminated hole is chosen over the four non-illuminated holes in the SP **(I)**. Note that the WM measures **(E–G)** represent fractions in which the number of correct CP responses **(H)** is normalized either to the sum of choice-pokes into the correct and incorrect illuminated hole (accuracy_lit_, **E**), the sum of choice-pokes into any of the five holes (accuracy_all_, **F**), or the number of CPs completed (% correct, **G**, including choice-pokes and omissions). Data is displayed as mean ± SEM; yellow line indicates chance level **(E)**. ^#^*p* < 0.1, *p < 0.05; **p < 0.01; ***p < 0.001, repeated-measures ANOVA, effects of group displayed on vertical lines, effects of block on horizontal lines, interactions indicated between data-lines.

To date, the T-maze test of rewarded alternation has been advanced as the primary assay of spatial WM (SWM) in rodents (Olton and Papas, 1979; Deacon and Rawlins, 2006; Kellendonk et al., 2009). However, it might have considerable drawbacks such as low trial numbers, lack of delay-independent challenges, of a DMTP-option (which is often used in humans and monkeys; e.g., Constantinidis and Goldman-Rakic, 2002), and of parameters to control for basic cued attention and motivation. Most importantly, it is confounded by the intrinsic preference of rodents for novel spaces, which is likely mediated by short-term habituation—a rather *passive* form of short-term memory based on the decrease of ascribed salience to sensory stimuli as they become more familiar (Barkus et al., 2014), see Sanderson and Bannerman (2012) for details of this argument. Rodents innately prefer to explore more novel over familiar spaces—such as the *correct* goal arm during the choice phase (CP) of the T-maze task (Figure 1A). The observation that mice typically perform well above chance level in both spontaneous alternation tasks and in the first trials in rewarded alternation (e.g., Bygrave et al., 2016) suggests that mice may rather use *passive* novelty-preference than an *active* intentional memory mechanism to solve it, because in these conditions there is no consolidated association between the win-shift strategy and reward (Sanderson and Bannerman, 2012). This confound is particularly problematic in schizophrenia research because short-term habituation is also impaired in schizophrenia (Holt et al., 2005; Barkus et al., 2014).

To solve many of these issues, operant paradigms of DMTP and delayed-non-matching-to-position (DNMTP) WM testing have been used (Dunnett, 1985; Pouzet et al., 1999; Barch et al., 2009; Smith et al., 2011). Therein rodents are typically presented with one retractable lever in the *sample phase (SP)*, which has to be pressed, and with two retractable levers in the *CP*, of which either the previously pressed one (DMTP) or the previously hidden one (DNMTP) has to be chosen to obtain the CP reward (Figure 1B). Equivalent paradigms have also been established using two poke-holes instead of two levers (Yhnell et al., 2016; Goto and Ito, 2017). However, these operant paradigms might have the problem—proposed for the classical primate SWM tasks (Castner et al., 2004)—that it can be solved by procedural long-term memory instead of WM: subjects can theoretically learn to encode the “correct choice” by positioning their body in front of the correct lever or poke-hole throughout the delay phase (Figure 1B).

To overcome the drawbacks of those T-maze and two-choice operant testing paradigms, we here develop a novel DMTP SWM assay for mice, using the layout of the operant box typically used for the 5-choice-serial-reaction-time task, 5-CSRTT [Bari et al., 2008; 5-choice based operant testing of SWM (5-CSWM), see Figure 1C]. Our assay builds upon the *combined attention and memory* (CAM) task previously developed for rats (Chudasama and Robbins, 2004; Chudasama et al., 2004).

## Materials and Methods

### Subjects

Twenty-four and 26 male C57BL/6J (Janvier, F) mice were used for initial establishment of the task and later assessment of training time in the optimized paradigm, respectively. Mice were 2–3 months old at the beginning of training and maintained under a 13:11 h light:dark schedule in enriched Typ II IVC cages (Tecniplast, I). All groups of mice were trained and tested in the light-phase and at the same time of day (±1 h), usually in the afternoon. All animal experiments conformed to the German Animal Rights Law (Tierschutzgesetz) 2013 and were approved by the Federal Ethical Review Committee of Baden-Württemberg (Regierungsprädisium Tübingen), Germany.

### Operant Working Memory Training Procedure

**Figure 2:**
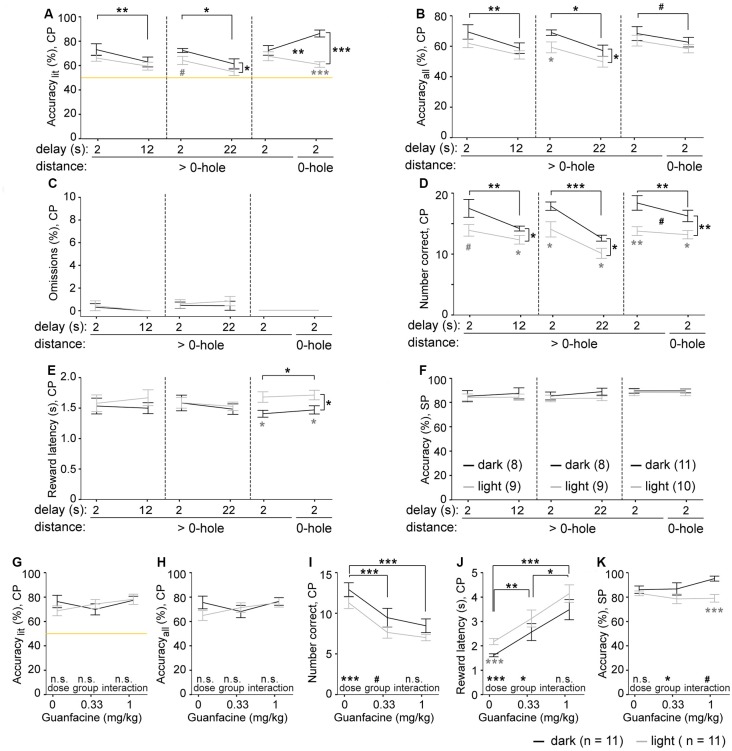
Effects of challenges and guanfacine on 5-CSWM performance. **(A–F)** Performance parameters for three distinct challenge paradigms and their individual baseline (averages of two sessions in each case), including an extension of the delay from 2 s to 12 s (left) and 22 s (middle), and the use of choice options that were always directly neighboring holes (right) instead of having 1–2 non-illuminated holes in between them. Parameters indicate SWM according to accuracy_lit_ **(A)** and accuracy_all_ **(B)**, calculated as in Figure 1, the relative number of CP omissions (normalized to number of CPs, **C**), the absolute number of correct CP responses **(D)**, the CP reward latency as control variable for motivation **(E)**, and the accuracy with which the illuminated hole was chosen in the SP as control variable for cue-directed attention **(F)**. Black significance indicators refer to repeated-measures ANOVA, effects of group displayed on vertical lines, effects of challenge on horizontal lines, interactions indicated between data-lines; gray stars indicate simple main-effects *post hoc* comparison between groups at individual conditions. *N*-numbers for groups trained in the *dark* (black) and *light* (gray) protocol are indicated in panel **(F)**. **(G–K)** Performance parameters for SWM, accuracy_lit_ **(G)** and accuracy_all_ **(H)**, as well as number of correct CP responses **(I)**, the CP reward latency as control variable for motivation **(J)**, and the accuracy with which the illuminated hole was chosen in the SP as control variable for cue-directed attention **(K)** are plotted for the groups tested in the *dark* (black) and *light* (gray) protocol after guanfacine pre-treatment at the indicated doses (n-numbers stated in legend underneath respective panels; 2 s SP-SD, 12 s delay). For clarity, statistical effects of drug-dose, group and interactions found in repeated-measures ANOVA are indicated above the respective words in each panel, while simple main-effects paired dose-comparisons are indicated on horizontal lines (black) and pairwise comparison between groups at individual doses are indicated below the data points (gray). n.s. or no indication *p* > 0.1, ^#^*p* < 0.1, *p < 0.05; **p < 0.01; ***p < 0.001. All data is shown as mean ± SEM.

In order to back-translate the spatial paradigm of WM testing in humans (Keefe et al., 1995) and primates (Arnsten et al., 1988; Friedman and Goldman-Rakic, 1988), avoiding the drawbacks of the T-maze (Figure 1A) and simple two-choice operant tasks (Figure 2B), we adapted the CAM task developed previously for rats (Chudasama and Robbins, 2004; Chudasama et al., 2004), which is based on the 5-CSRTT and conducted in a specialized 5-choice operant box (Med Associates, VT, USA). The 5-CSWM task flow (Figure 1C), involves a *SP* in which the mice have to poke into an illuminated hole in the 5-choice wall, a *delay phase, DP* during which the mice have to return to the opposite wall and poke into the illuminated reward receptacle, and a *CP* in which the mice are presented with two illuminated holes of which they have to choose either the one that was *illuminated* in the prior SP (DMTP) or the *other* one (DNMTP). In contrast to the original CAM-task, which was trained in a two-step procedure with an initial 5-CSRTT acquisition (SP only) requiring 6–8 months for the full training in rats (Chudasama et al., 2005), we trained mice in the full sequence, including both phases, from the beginning of SWM training. Thereby mice obtained a small and decreasing reward for correct target detection in the SP and a large reward for a correct CP response (Figures 1C,D). The main stages of the training differed by the distance between the two holes illuminated in the CP. Mice transitioned to the next stage, if—in three consecutive daily 30 min sessions—the accuracy with which they chose the correct over the incorrect illuminated hole (termed accuracy_lit_) was ≥70% and the number of correct choices was ≥10. Within those main stages, mice were advanced across sub-stages relating to the SP: whenever ≥25 correct SP choices have been made in three consecutive sessions, the SP reward was decreased to 10 μl and then 0 μl in main stages 1 and 2, or the stimulus-presentation time (stimulus duration, SD) was reduced to 8 s and then 4 s in main stage 3 (Figure 1D); see Supplementary Methods and the task stage overview in Supplementary Table S2 for further details. All raw data are available from the corresponding author at reasonable request.

## Results

### Establishment of a 5-Choice-Based Operant Working Memory Task in Mice

For initial implementation of the operant SWM procedure, two subgroups of 12 mice each were trained with a default state of an *illuminated* house light (i.e., light only switched off for time-outs after erroneous actions; subsequently termed “*light* protocol” (following the murine 5-CSRTT, e.g., Bygrave et al., 2016)—one group in the DMTP, the other in the DNMTP paradigm. We compared key parameters of training progress between the first and the third block of seven training sessions (Figures 1E–I; see Supplementary Table S1 for statistical details on this and all subsequent data). Both groups improved significantly in the *SP* accuracy (choosing the illuminated over the four non-illuminated holes; Figure 1I). In the* CP*, however, the DNMTP-group still performed at chance level in the key working-memory measure *accuracy*_lit_, while the DMTP-group was consistently higher in both phases (Figure 1E, see also Supplementary Figure S1). Surprisingly, however, even the DMTP-group did not improve in WM accuracy *between* these two early training blocks (Figures 1E,F), but only in the absolute and relative number of correct CP responses (Figures 1G,H), indicating a combination of decreased omissions and higher preference for poking illuminated over non-illuminated holes. The DNMTP group was stopped and re-purposed for training with an inverted house-light schedule (*dark* protocol) beginning with the five-hole habituation, followed by the DMTP paradigm.

### Increasing Delay Reduces Working Memory Performance in Wildtype Mice

We trained both subgroups to the final stage 3 with a SD in the CP of 4 s (see Figure 1D) using either the *light* (*n* = 9; 160 training sessions) or the *dark* protocol (*n* = 8; 110 training sessions) before assessing the delay-dependence of WM performance with challenge protocols (four other mice only reached the final stage 30 sessions later and were tested only on the last, non-delay challenge, see below; two mice did not reach the final stage at all). We conducted three test sequences of 4 days each, whereby during the first 2 days the mice were trained on the baseline protocol (2 s delay), while on the last 2 days the mice were exposed to a challenge condition. During the first challenge, the delay phase was extended to 12 s, in the second challenge to 22 s, and in the third challenge the delay remained 2 s, but the two choice options were neighboring holes (no gap-hole), and therefore more similar to one another.

We found that the increase of the delay significantly worsened accuracy in the WM component of the task (Figures 2A,B), but not in the attentional SP parameter (Figure 2F). Surprisingly, CP omissions were extremely rare, with group averages below 1%, whereby most animals showed no omissions at all, regardless of protocol (Figure 2C). This contrasts greatly with SP omission rates which were consistently between 50% and 57% on average, in each testing condition (not shown), consistent with experience from the 5-CSRTT in mice (e.g., Grimm et al., 2018). The number of CP correct responses decreased with both delay challenges (Figure 2D), likely reflecting a combination of longer trials and lower WM accuracy, but not decreased motivation, as indicated by constant average reward latencies (Figure 2E). Interestingly though, mice tested in the *dark* protocol showed more correct responses (Figure 2D), and partly also higher WM accuracy (Figures 2A,B) than the subgroup subjected to the *light* protocol.

Surprisingly, the third challenge paradigm of presenting neighboring holes as choice options did *not* decrease WM performance; in fact, in the group trained in the *dark* protocol accuracy_lit_ even increased, leading to a significant challenge-group interaction (Figure 2A).

### Guanfacine Is Largely Ineffective in Operant DMTP Working Memory in Mice

Next, we assessed the effect of guanfacine—a candidate drug to improve WM in some tests in humans (Jäkälä et al., 1999a) and monkeys (Arnsten et al., 1988; Franowicz and Arnsten, 1998)—on performance in the rodent 5-CSWM paradigm. Guanfacine (0.33 and 1 mg/kg) did not increase WM accuracy when analyzing both paradigms (*dark* and *light* protocol, 12 s delay, 2 s SP SD) combined (Figures 2G,H). However, when regarding the *light* subgroup alone, there was a trend (*p* = 0.065) for an improvement in a repeated-measures ANOVA and a significant improvement when comparing performance under 1 mg/kg vs. vehicle in the measure accuracy_all_ (*p* = 0.033, *t-test*; *p* = 0.085 for accuracy_lit_). Furthermore, we could confirm the general efficacy of the drug due to a highly significant dose-dependent increase of the reward latency and a decrease of correct and premature responses (Figures 2I,J, Supplementary Figure S3), in line with its effect on the 5-CSRTT in mice described previously (Pillidge et al., 2014). Interestingly, the drug also led to a qualitative divergence of the SP accuracy between the two subgroups of mice, suggesting that attention is affected in dependence on the illumination state (or: visibility of potentially distracting stimuli) in the operant box (*p* = 0.013 for an effect of group, *p* = 0.092 for a group-dose interaction, repeated-measures ANOVA; Figure 2K). We similarly assessed modafinil (15 and 30 mg/kg) and the mGluR5-positive allosteric modulator LSN 2463359 (0.33–10 mg/kg), but found no effect on SWM performance (Supplementary Figure S2).

### Training Demand in the *Dark* DMTP Protocol

**Figure 3:**
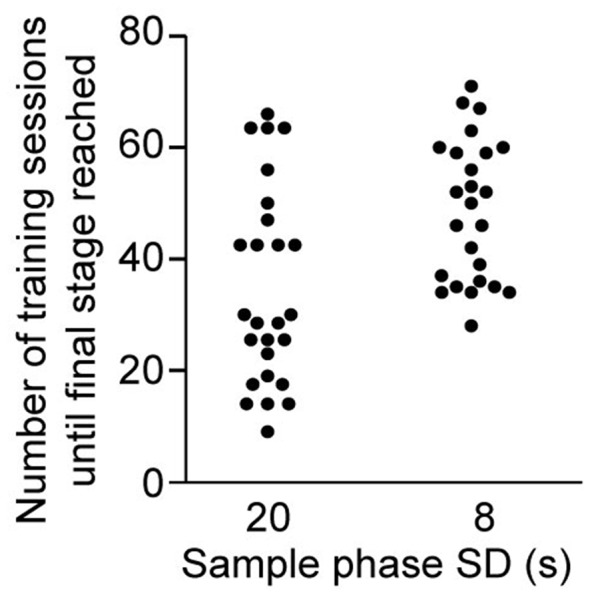
Training demand for DMTP *dark* protocol. Number of daily 30-min training sessions required to transition to stage 3 (see Figure 1D) with an SD of 20 s or 8 s, as indicated (*n* = 26, of which one mouse did not reach the 8 s—SD substage within 75 sessions).

Given that performance seemed to be highest in the *dark* *DMTP* protocol compared to other conditions tested, we trained a separate cohort in this protocol to estimate the number of sessions required to reach the baseline stage (3) following the fully established training schedule (Figure 1D, see “Materials and Methods” section). We found that the *median* and *maximum* number of daily training sessions needed to reach the baseline stage 3 with an SD of 8 s were 50 and 71 (*n* = 26, of which one mouse failed to reach the SD 8 s sub-stage within 75 sessions; Figure 3; habituation sessions not counted).

### Potential Mediation Strategies

In the 5-CSWM task, the requirement for the animal to return to the opposite wall ensures that the mouse cannot simply position its body in front of the correct choice option early during the delay phase (as a mediating strategy). But this measure becomes the less effective the longer the delay is. With delays of 10 or 20 s, the time requirement for the transition between the opposite walls of the operant box is relatively small compared to the total delay time, and therefore the amount of time that the animal can sit in front of the correct hole, thereby encoding the correct choice by its body position, is relatively large. A premature response into the correct hole may indicate this mediating strategy, as the mice would only perform such a premature response, when sitting in front of the correct hole during the delay phase; additionally, such unpunished poking might serve as a rehearsal to refresh the memory. We evaluated this possibility during the guanfacine experiment, where animals are faced with an extended delay of 10 s and where premature responding would likely be changed given the effect of guanfacine on this parameter in the 5-CSRTT (Pillidge et al., 2014). We found, that—under vehicle conditions—mice indeed made *correct* premature responses in about one-third of the CP trials (Supplementary Figure S3). However, they also made *incorrect* premature responses in about half of the CP trials in the *light* protocol and one-third of the CP trials in the *dark* protocol (Supplementary Figure S3). Under vehicle conditions and across animals, accuracy_lit_ was not correlated to the relative number of correct premature responses (*dark* protocol: *r* = −0.01, *p* = 0.978; *light* protocol: *r* = 0.11, *p* = 0.759). Furthermore, premature responding in the delay phase and WM performance could be dissociated by guanfacine treatment: while the WM accuracy remained constant (*dark* protocol) or increased qualitatively (*light* protocol) with increasing dose of guanfacine (Figure 2G), the number of premature responses declined sharply and significantly (Supplementary Figure S3). These observations show, that premature poking into the correct hole does not serve as a necessary or consistent strategy to mediate WM performance.

## Discussion

The presented results demonstrate that mice can acquire a simplified version of the CAM task (Chudasama et al., 2005) within *ca*. 3 months of daily training allowing the assessment of DMTP WM in an operant paradigm. The 5-CSWM design has several advantages over prior SWM paradigms, including: (a) it is not confounded by relative spatial novelty of the choice options; (b) mice cannot easily encode the correct choice option by the position of their body during the delay phase, because they have to return to the opposite wall, and—additionally—because the set of choice options and their spatial configuration varies greatly from trial to trial and can involve very nearby stimuli; (c) confounds of reduced sustained attention and motivation can be controlled for using the variables of SP accuracy and CP reward latency; and (d) beyond increases of the delay, WM could *theoretically* be challenged in multiple other ways, e.g., using distractions or providing more choice options—which remain to be explored. Importantly, however, animals were not able to perform above the chance level when trained in the DNMTP paradigm in the 5-CSWM task—at least not within 21 sessions of training. The possibility of using a touch-screen paradigm to realize a DNMTP equivalent of the 5-CSWM, as previously done in rats (McAllister et al., 2013), remains to be determined, however.

A comparison between our results and previously published murine poke-hole-based operant WM paradigms highlights the potential role of mediation strategies [see (b) above]: seemingly similar to our paradigm, Yhnell et al. (2016) used a 9-choice nose-poke-wall to present two choice stimuli at a time and not only observed a much faster training progress (achieving an average accuracy of *ca*. 70% in eight sessions) but also demonstrated that mice could acquire a DNMTP paradigm of the same task. Importantly though, mice were not required to turn to the opposite wall during the delay phase (they only needed to poke once into the nearby middle hole of the 9-choice wall) and the choice configuration was identical across trials and sessions (holes 3 and 7). Therefore, the mediation strategy of encoding the correct choice option with the position of their body as described above (Figure 1B) is more likely to occur and is probably a key factor that allowed mice to acquire the DNMTP-paradigm in that case. Another study reported the possibility of training mice in a DNMTP-paradigm, that emulated the 2-lever paradigm (Figure 2B) with two nose-poke holes positioned on either side of the receptacle but requiring the animal to poke into an additional hole at the opposite wall during the delay phase (Goto and Ito, 2017). Mice achieved an average accuracy of *ca*. 70% already by the third training session. Despite the requirement for shuttling between the walls, the acquisition of a habit of circling through the box by 270° towards the opposite side after the SP—poking into the third hole *en route—*could have served as a mediating strategy as it allows to solve the task without engaging WM. Again, this could explain the stark difference in training progress compared to our data and might illustrate the value of using varying choice configurations such that the effective usage of habitual movements to solve the task is prevented.

In addition to the generally high difficulty of the 5-CSWM task, presumably resulting from a lack of mediation strategies that can aid task acquisition, one would expect the applied DNMTP paradigm to be more difficult to the equivalent DMTP version, due to the additional demand for manipulating the information held in memory (Baddeley, 1992) to generate an action that is distinct from the action performed in the prior SP.

At the same time, the lack of a challenging effect (*light* protocol) or even an increase in performance (*dark* protocol) seen with the proximity challenge is surprising. This challenge was expected to increase task difficulty by making the choice options more similar (as they are very nearby). But the observed lack of a decrease in task performance is consistent with what has been reported in rats performing the CAM-task, where no relation between proximity between choice holes and choice accuracy has been found (Chudasama et al., 2004). This opens the possibility to stage the training process differently, as it is rather the number of possible choice configurations than the proximity between holes that increases the difficulty of the task. Therefore, in the early stages, the number of choice-configurations should be kept low, but holes can already be in close proximity.

It should also be noted that—in contrast to the original CAM-task (Chudasama and Robbins, 2004; Chudasama et al., 2004)—the paradigm presented here does not involve very short (≤1 s) SP SDs as required to challenge sustained attention nor did we assess premature responding (impulsivity) by increasing the waiting time before SP-onset. Instead, our current protocol focuses solely on WM. However, testing with a 2 s SP-SD (experiments with modafinil and guanfacine) showed a similar SP accuracy as when tested with a 4 s SP-SD (compare for example Figure 2F vs. Figure 2K), suggesting that challenging sustained attention by a further SD reduction should be possible also in mice.

We furthermore showed that guanfacine and modafinil did not enhance SWM in this 5-CSWM task at the tested doses and delays—in contrast to their effect on T-maze performance in rodents (Béracochéa et al., 2001; Franowicz et al., 2002). This was not due to a general lack of efficacy of the tested doses, because significant changes of reward latencies were observed in the expected direction (increase by guanfacine, Pillidge et al., 2014, decrease by modafinil, Young et al., 2011). This could be taken as an indication of a limitation of the translational value of the 5-CSWM. However, it should be noted that the WM-enhancing effects of these compounds in humans are disputed as well. Both drugs show limited efficacy in schizophrenia (Friedman et al., 2001; Millan et al., 2012; Scoriels et al., 2013). But also in healthy humans, a considerable number of studies have failed to see WM-improvement by modafinil in non-sleep-deprived subjects (Battleday and Brem, 2015) or by guanfacine in a DMTP WM paradigm (Jäkälä et al., 1999b). Also, the actual physiological and psychological mechanism(s) of modafinil’s cognition-enhancing action remains unclarified, as several neurotransmitter systems, including all major mono-aminergic neuromodulators, are affected by this compound and increase of wakefulness (instead of direct WM-improvement) may be a key mediator of its nootropic action (Murillo-Rodríguez et al., 2018; Sahakian and Savulich, 2019). However, the significance of our current pharmacological results is clearly limited as an extended dose-range and different challenges and disease models with impaired WM remain to be assessed in the 5-CSWM, especially for modafinil (Béracochéa et al., 2001; Piérard et al., 2006, 2007; Murphy et al., 2015). Furthermore, our testing of LSN 2463359 in this task remains inconclusive, as none of the measured behavioral variables showed any dose-dependent effect. This questions the general efficacy of the tested doses, although the same dose-range showed efficacy in rats before (Gastambide et al., 2013; Gilmour et al., 2013).

We envision that the murine 5-CSWM task will help to determine the neural circuit basis of DMTP SWM and aid drug discovery for currently pharmacoresistant WM impairments.

## Data Availability

All datasets generated for this study are included in the manuscript and/or the Supplementary Files.

## Ethics Statement

### Animal Subjects

The animal study was reviewed and approved by Regierungspraesidium Tuebingen, Germany.

## Author Contributions

JT and DK designed the study, analyzed data and wrote the manuscript. JT conducted all experiments.

## Conflict of Interest Statement

The authors declare that the research was conducted in the absence of any commercial or financial relationships that could be construed as a potential conflict of interest.

## References

[B1] Arnsten A. F. Cai J. X. Goldman-Rakic P. S. (1988). The α-2 adrenergic agonist guanfacine improves memory in aged monkeys without sedative or hypotensive side effects: evidence for α-2 receptor subtypes. J. Neurosci. 8, 4287–4298. 10.1523/JNEUROSCI.08-11-04287.1988 2903226 PMC6569464

[B2] Baddeley A. (1992). Working memory. Science 255, 556–559. 10.1126/science.1736359 1736359

[B3] Baddeley A. D. Hitch G. (1974). “Working memory,” in Psychology of Learning and Motivation, ed. Bower G. H. (New York, NY: Academic Press), 47–89.

[B5] Barch D. M. Berman M. G. Engle R. Jones J. H. Jonides J. MacDonald A. III.. (2009). CNTRICS final task selection: working memory. Schizophr. Bull. 35, 136–152. 10.1093/schbul/sbn153 18990711 PMC2643954

[B4] Barch D. M. Smith E. (2008). The cognitive neuroscience of working memory: relevance to CNTRICS and schizophrenia. Biol. Psychiatry 64, 11–17. 10.1016/j.biopsych.2008.03.003 18400207 PMC2483314

[B6] Bari A. Dalley J. W. Robbins T. W. (2008). The application of the 5-choice serial reaction time task for the assessment of visual attentional processes and impulse control in rats. Nat. Protoc. 3, 759–767. 10.1038/nprot.2008.41 18451784

[B7] Barkus C. Sanderson D. J. Rawlins J. N. P. Walton M. E. Harrison P. J. Bannerman D. M. (2014). What causes aberrant salience in schizophrenia? A role for impaired short-term habituation and the GRIA1 (GluA1) AMPA receptor subunit. Mol. Psychiatry 19, 1060–1070. 10.1038/mp.2014.91 25224260 PMC4189912

[B8] Battleday R. M. Brem A.-K. (2015). Modafinil for cognitive neuroenhancement in healthy non-sleep-deprived subjects: a systematic review. Eur. Neuropsychopharmacol. 25, 1865–1881. 10.1016/j.euroneuro.2015.07.028 26381811

[B9] Béracochéa D. Cagnard B. Célérier A. le Merrer J. Pérès M. Piérard C. (2001). First evidence of a delay-dependent working memory-enhancing effect of modafinil in mice. Neuroreport 12, 375–378. 10.1097/00001756-200102120-00038 11209953

[B10] Bygrave A. M. Masiulis S. Nicholson E. Berkemann M. Sprengel R. Harrison P.. (2016). Knockout of NMDA-receptors from parvalbumin interneurons sensitizes to schizophrenia-related deficits induced by MK-801. Transl. Psychiatry 6:e778. 10.1038/tp.2016.44 27070406 PMC4872402

[B11] Castner S. A. Goldman-Rakic P. S. Williams G. V. (2004). Animal models of working memory: insights for targeting cognitive dysfunction in schizophrenia. Psychopharmacology 174, 111–125. 10.1007/s00213-003-1710-9 15205882

[B13] Chudasama Y. Dalley J. W. Nathwani F. Bouger P. Robbins T. W. (2004). Cholinergic modulation of visual attention and working memory: dissociable effects of basal forebrain 192-IgG-saporin lesions and intraprefrontal infusions of scopolamine. Learn. Mem. 11, 78–86. 10.1101/lm.70904 14747520 PMC321317

[B14] Chudasama Y. Nathwani F. Robbins T. W. (2005). d-Amphetamine remediates attentional performance in rats with dorsal prefrontal lesions. Behav. Brain Res. 158, 97–107. 10.1016/j.bbr.2004.08.011 15680198

[B12] Chudasama Y. Robbins T. W. (2004). Dopaminergic modulation of visual attention and working memory in the rodent prefrontal cortex. Neuropsychopharmacology 29, 1628–1636. 10.1038/sj.npp.1300490 15138446

[B15] Constantinidis C. Goldman-Rakic P. S. (2002). Correlated discharges among putative pyramidal neurons and interneurons in the primate prefrontal cortex. J. Neurophysiol. 88, 3487–3497. 10.1152/jn.00188.2002 12466463

[B16] Deacon R. M. J. Rawlins J. N. P. (2006). T-maze alternation in the rodent. Nat. Protoc. 1, 7–12. 10.1038/nprot.2006.2 17406205

[B17] Dunnett S. B. (1985). Comparative effects of cholinergic drugs and lesions of nucleus basalis or fimbria-fornix on delayed matching in rats. Psychopharmacology 87, 357–363. 10.1007/bf00432721 3936093

[B18] Franowicz J. S. Arnsten A. F. T. (1998). The α-2a noradrenergic agonist, guanfacine, improves delayed response performance in young adult rhesus monkeys. Psychopharmacology 136, 8–14. 10.1007/s002130050533 9537677

[B19] Franowicz J. S. Kessler L. E. Borja C. M. D. Kobilka B. K. Limbird L. E. Arnsten A. F. T. (2002). Mutation of the α2A-adrenoceptor impairs working memory performance and annuls cognitive enhancement by guanfacine. J. Neurosci. 22, 8771–8777. 10.1523/JNEUROSCI.22-19-08771.2002 12351753 PMC6757781

[B20] Friedman H. R. Goldman-Rakic P. S. (1988). Activation of the hippocampus and dentate gyrus by working-memory: a 2- deoxyglucose study of behaving rhesus monkeys. J. Neurosci. 8, 4693–4706. 10.1523/JNEUROSCI.08-12-04693.1988 3199202 PMC6569568

[B21] Friedman J. I. Adler D. N. Temporini H. D. Kemether E. Harvey P. D. White L.. (2001). Guanfacine treatment of cognitive impairment in schizophrenia. Neuropsychopharmacology 25, 402–409. 10.1016/s0893-133x(01)00249-4 11522468

[B22] Gastambide F. Gilmour G. Robbins T. W. Tricklebank M. D. (2013). The mGlu5 positive allosteric modulator LSN2463359 differentially modulates motor, instrumental and cognitive effects of NMDA receptor antagonists in the rat. Neuropharmacology 64, 240–247. 10.1016/j.neuropharm.2012.07.039 22884612

[B23] Gilmour G. Broad L. M. Wafford K. A. Britton T. Colvin E. M. Fivush A.. (2013). *In vitro* characterisation of the novel positive allosteric modulators of the mGlu5 receptor, LSN2463359, and LSN2814617 and their effects on sleep architecture and operant responding in the rat. Neuropharmacology 64, 224–239. 10.1016/j.neuropharm.2012.07.030 22884720

[B24] Goto K. Ito I. (2017). The asymmetry defect of hippocampal circuitry impairs working memory in β2-microglobulin deficient mice. Neurobiol. Learn. Mem. 139, 50–55. 10.1016/j.nlm.2016.12.020 28039089

[B25] Grimm C. M. Aksamaz S. Schulz S. Teutsch J. Sicinski P. Liss B.. (2018). Schizophrenia-related cognitive dysfunction in the Cyclin-D2 knockout mouse model of ventral hippocampal hyperactivity. Transl. Psychiatry 8:212. 10.1038/s41398-018-0268-6 30301879 PMC6178344

[B26] Holt D. J. Weiss A. P. Rauch S. L. Wright C. I. Zalesak M. Goff D. C.. (2005). Sustained activation of the hippocampus in response to fearful faces in schizophrenia. Biol. Psychiatry 57, 1011–1019. 10.1016/j.biopsych.2005.01.033 15860342

[B27] Jäkälä P. Riekkinen M. Sirviö J. Koivisto E. Kejonen K. Vanhanen M.. (1999a). Guanfacine, but not clonidine, improves planning and working memory performance in humans. Neuropsychopharmacology 20, 460–470. 10.1016/s0893-133x(98)00127-4 10192826

[B28] Jäkälä P. Sirviö J. Riekkinen M. Koivisto E. Kejonen K. Vanhanen M.. (1999b). Guanfacine and clonidine, α 2-agonists, improve paired associates learning, but not delayed matching to sample, in humans. Neuropsychopharmacology 20, 119–130. 10.1016/s0893-133x(98)00055-4 9885792

[B29] Keefe R. S. E. Roitman S. E. Harvey P. D. Blum C. S. DuPre R. L. Prieto D. M.. (1995). A pen-and-paper human analogue of a monkey prefrontal cortex activation task: spatial working memory in patients with schizophrenia. Schizophr. Res. 17, 25–33. 10.1016/0920-9964(95)00027-j 8541247

[B30] Kellendonk C. Simpson E. H. Kandel E. R. (2009). Modeling cognitive endophenotypes of schizophrenia in mice. Trends Neurosci. 32, 347–358. 10.1016/j.tins.2009.02.003 19409625 PMC4928481

[B31] McAllister K. A. L. Saksida L. M. Bussey T. J. (2013). Dissociation between memory retention across a delay and pattern separation following medial prefrontal cortex lesions in the touchscreen TUNL task. Neurobiol. Learn. Mem. 101, 120–126. 10.1016/j.nlm.2013.01.010 23396186 PMC3757163

[B32] Millan M. J. Agid Y. Brüne M. Bullmore E. T. Carter C. S. Clayton N. S.. (2012). Cognitive dysfunction in psychiatric disorders: characteristics, causes and the quest for improved therapy. Nat. Rev. Drug Discov. 11, 141–168. 10.1038/nrd3628 22293568

[B33] Murillo-Rodríguez E. Barciela Veras A. Barbosa Rocha N. Budde H. Machado S. (2018). An overview of the clinical uses, pharmacology, and safety of modafinil. ACS Chem. Neurosci. 9, 151–158. 10.1021/acschemneuro.7b00374 29115823

[B34] Murphy H. M. Ekstrand D. Tarchick M. Wideman C. H. (2015). Modafinil as a cognitive enhancer of spatial working memory in rats. Physiol. Behav. 142, 126–130. 10.1016/j.physbeh.2015.02.003 25656691

[B35] Olton D. S. Papas B. C. (1979). Spatial memory and hippocampal function. Neuropsychologia 17, 669–682. 10.1016/0028-3932(79)90042-3 522981

[B36] Piérard C. Liscia P. Philippin J.-N. Mons N. Lafon T. Chauveau F.. (2007). Modafinil restores memory performance and neural activity impaired by sleep deprivation in mice. Pharmacol. Biochem. Behav. 88, 55–63. 10.1016/j.pbb.2007.07.006 17698177

[B37] Piérard C. Liscia P. Valleau M. Drouet I. Chauveau F. Huart B.. (2006). Modafinil-induced modulation of working memory and plasma corticosterone in chronically-stressed mice. Pharmacol. Biochem. Behav. 83, 1–8. 10.1016/j.pbb.2005.11.018 16439006

[B38] Pillidge K. Porter A. J. Dudley J. A. Tsai Y.-C. Heal D. J. Stanford S. C. (2014). The behavioural response of mice lacking NK1 receptors to guanfacine resembles its clinical profile in treatment of ADHD. Br. J. Pharmacol. 171, 4785–4796. 10.1111/bph.12860 25074741 PMC4209942

[B39] Pouzet B. Welzl H. Gubler M. K. Broersen L. Veenman C. L. Feldon J.. (1999). The effects of NMDA-induced retrohippocampal lesions on performance of four spatial memory tasks known to be sensitive to hippocampal damage in the rat. Eur. J. Neurosci. 11, 123–140. 10.1046/j.1460-9568.1999.00413.x 9987017

[B40] Sahakian B. J. Savulich G. (2019). Innovative methods for improving cognition, motivation and wellbeing in schizophrenia. World Psychiatry 18, 168–170. 10.1002/wps.20649 31059610 PMC6502422

[B41] Sanderson D. J. Bannerman D. M. (2012). The role of habituation in hippocampus-dependent spatial working memory tasks: evidence from GluA1 AMPA receptor subunit knockout mice. Hippocampus 22, 981–994. 10.1002/hipo.20896 21125585 PMC3490380

[B42] Scoriels L. Jones P. B. Sahakian B. J. (2013). Modafinil effects on cognition and emotion in schizophrenia and its neurochemical modulation in the brain. Neuropharmacology 64, 168–184. 10.1016/j.neuropharm.2012.07.011 22820555

[B43] Smith J. W. Gastambide F. Gilmour G. Dix S. Foss J. Lloyd K.. (2011). A comparison of the effects of ketamine and phencyclidine with other antagonists of the NMDA receptor in rodent assays of attention and working memory. Psychopharmacology 217, 255–269. 10.1007/s00213-011-2277-5 21484239

[B44] Yhnell E. Dunnett S. B. Brooks S. P. (2016). The utilisation of operant delayed matching and non-matching to position for probing cognitive flexibility and working memory in mouse models of Huntington’s disease. J. Neurosci. Methods 265, 72–80. 10.1016/j.jneumeth.2015.08.022 26321735 PMC4863528

[B45] Young J. W. Kooistra K. Geyer M. A. (2011). Dopamine receptor mediation of the exploratory/hyperactivity effects of modafinil. Neuropsychopharmacology 36, 1385–1396. 10.1038/npp.2011.23 21412225 PMC3096808

