# Supplementary material for: Operant Assessment of DMTP Spatial Working Memory in Mice
Source: Front Behav Neurosci. 2019 Aug 27;13:193. doi: 10.3389/fnbeh.2019.00193 (PMC6718719; doi:10.3389/fnbeh.2019.00193)
Supplement: Supplementary file 1 [file Data_Sheet_1.docx]

Supplementary Information

# Operant assessment of DMTP spatial working memory in mice

***Running title:*** Operant working memory testing in mice

***Keywords:*** spatial working memory, delayed-matching-to-position, guanfacine, modafinil, combined attention and memory (CAM) task

***Authors:***

Jasper Teutsch^1,2^, Dennis Kätzel, D.Phil^1,^*

^1^ Institute of Applied Physiology, Ulm University, Ulm, Germany

^2^ *Present address:* ETH Zürich, Zürich, Switzerland

*** Correspondence:**

Dennis Kätzel, Institute of Applied Physiology, Ulm University, 89081 Ulm, Germany; dennis.kaetzel@uni-ulm.de

# Supplementary Methods

## Animal models

All experiments were performed in accordance with institutional guidelines, the German Animal Rights Law (Tierschutzgesetz, 2013) and were approved by the Regierungspräsidium Tübingen, Germany. All mice were on a C57BL/6J background. 24 and 26 male C57BL/6J (Janvier, F) mice were used for initial establishment of the task and later assessment of training time in the optimized paradigm, respectively. Mice were 2-3 mo old at the beginning of training and maintained under a 13:11 hours light:dark schedule in enriched Typ II IVC cages (Tecniplast, I) in a temperature and humidity controlled vivarium. IVC cages containing sawdust, sizzle nest and one cardboard house as enrichment were used as homecages. Animals were always group-housed. Water was provided *ad libitum* at all times. All experiments were conducted during the light phase.

## 5-CSWM task based operant working memory training for mice

### Habituation stages

Before starting training, animals were removed from *ad libitum* feeding and put on a food deprivation schedule. Weight was kept at 85-95% of individually determined average free feeding weights (average weight across 3 d before food-scheduling started). Animals were accustomed to the future strawberry milk reward (Müller®, G; nutritional values closely matching Yazoo® strawberry milkshake often used for the 5-CSRTT) by providing it 2-3 times in their home cages before food-removal.

On the first 1-2 d after food-removal, animals were put in the operant boxes to accustom them to this new environment (Med Associates Inc., US; ENV-307A-CT chambers incl. ENV-307A-GF stainless steel grid floor, with ENV-115C 5-choice wall on one side and ENV-303RMW dual pellet/liquid receptacle with 18ga pipe, ENV-303HDW Head entry detector and ENV-302RL-1 receptacle light centred on the opposite wall; the reward was provided by a PHM-100A syringe pump). Doors were closed, the fan and receptacle light turned on, the house light was set into the state which was to be used during later training stages (*dark* or *light* protocol), and the reward receptacle was illuminated and filled with strawberry milk. The mice were allowed 10 min to drink the milk. The receptacle was refilled, if emptied within this time. If mice did not drink in the first session, this procedure was repeated once on the same day. If mice did not drink on the first day at all, the described procedure was repeated on the following day. Mice were only advanced to the next stage, if they consumed the milk reward inside the box.

Next, mice were trained in the basic operant cycle of poking holes in the 5-choice wall in order to obtain a milk reward. The fan was turned on throughout, the house light was set into the state which was to be used during later training stages (*dark* or *light* protocol) and all five holes were illuminated initially. When a poke into one of the holes on the 5-choice wall was registered, hole lights turned off, a 20 µl reward was provided in the receptacle and the receptacle light was turned on, simultaneously. 2 s after the mouse entered the receptacle to obtain the reward, the receptacle light was turned off again, signaling the mouse that the time to drink is over and a new trial starts, i.e. all lights of the 5-choice wall were illuminated again. The time period for which the 5-choice-wall lights remained illuminated (stimulus duration, SD) was unlimited. The number of pokes into illuminated holes (equal to the number of earned rewards) was recorded and needed to be at least 40 on three consecutive days before the actual working memory training was started in stage 1on the next day (see Figure 1D for the stages of the task).

### 5-CSWM task design

One complete trial of the 5-choice-based operant spatial working memory task (5-CSWM) consisted of two phases (see Figure 1C): In the **sample phase** (SP) one random hole of the 5-choice wall was illuminated for a given amount of time (stimulus duration, SD; see below). To complete the sample trial, the mouse had to poke into this sample hole within a given time period (limited hold, LH) which included the SD but exceeded it by 2 s. If, in a given time period, no poke (omission) or an incorrect poke (into a non-illuminated hole) was registered, the current trial was stopped, a 5 s time-out (“punishment”) period (alteration of regular house light state), and the regular 15 s inter-trial interval (ITI) followed, and then a new trial was initiated. After a correct poke, however, the receptacle light on the opposite wall was switched on to signal that the mouse had to approach that wall and poke into the receptacle. In the initial stages (see Figure 1C), this response was incentivized by simultaneous delivery of a small milk reward (20 μl or 10 μl). After activation of the receptacle break beam, the **delay phase** was initiated (2 s during training; 12 or 22 s for delay challenges), which was immediately followed by the choice phase. In the **choice phase** (CP) the mouse is presented with two illuminated holes, one of them being the same as the one presented in the sample trial. To successfully complete the **choice phase**, the mouse had to poke either into the hole that was already illuminated in the sample trial (DMTP paradigm) or into the other one (DNMTP). When choosing the correct hole, the lights on the 5-choice wall were switched off, the receptacle light was illuminated, and a milk reward (60 μl) delivered. After an ITI of 15 s a new trial was initiated. If in a given time period the mouse gave an incorrect or no response at all (omission), the current trial was stopped and a 5 s time-out period (no reward given) was applied, followed by a 15 s ITI, before a new trial (SP) was initiated. One training session lasted at least 30 min, but would continue slightly beyond that, if the ongoing trial still had to be completed. The choice of the holes was determined randomly, so that all SP and CP configurations were presented with equal probability. Note that – similarly to the rat CAM task (Chudasama et al., 2005) - premature responses were never punished and did not result in an interruption of the trial.

Assessed output measures were:

| Phase | Parameter | Definition |
| --- | --- | --- |
| Sample | #correct | # of pokes into the lit hole |
|  | Response latency (correct or incorrect) | Time span between start of stimulus and first poke |
|  | Accuracy | Choice frequency of the sample hole against all other (non-illuminated) holes |
|  | %correct | Choice frequency of the correct sample hole against the total amount of trials |
|  | %omissions | Percentage of trials without a decision in the provided time relative to the total amount of trials |
| Choice | Response latency (correct or incorrect) | Time span between start of stimulus and first poke |
|  | Reward latency | Time span between first poke and first receptacle beam break |
|  | Accuracy_lit_ | Choice frequency of the sample hole against the additionally lit hole |
|  | Accuracy_all_ | Choice frequency of the sample hole against all other (illuminated and non-illuminated) holes |
|  | #correct | # of pokes into the correct hole |
|  | %correct | Choice frequency of the correct hole against the total amount of trials |
|  | %omissions | Percentage of trials without a decision in the provided time relative to the total amount of trials |

### 5-CSWM task training

The **three main stages of the 5-CSWM** task were primarily characterized by the distance (number of non-illuminated holes) between the two holes presented in the choice phase – 2-hole gap in stage 1, 1-hole gap in stage 2, and either 1- or 2-hole gaps (randomly) in stage 3. For the third challenge protocol (“proximity challenge”) this distance was further reduced to 0, i.e. the two choice holes were immediately neighbouring. The implies that – when taking the identity of the *correct* hole into account - there are 4 possible choice configurations in stage 1, 6 in stage 2, 10 in stage 3 and 8 in the proximity challenge. The assumption is, that the difficulty of the task and the difficulty to solve it by positioning the body in front of the correct hole during the delay increases with the number of expectable choice options as well as with decreasing distance (higher similarity) between the two choice holes. However, task difficulty did not seem to increase with decreasing distance, as indicated by the results of the proximity challenge (Figure 2A-F). (Note that - based on the result of the proximity challenge - a further modification, that could be made in future studies to achieve the same goal, would be to illuminate more than 2 holes, so that 2 or more incorrect holes would be presented simultaneously with the correct one in the CP, lowering the probability to pick the correct hole by chance or to encode the correct choice by positioning the body on either side of the box. Also note, that the observation, that mice trained in the *dark* protocol are – if anything – better than those trained in the *light* protocol (Figure 2A-F), suggests, that mice do not use orientation and body-placement according to visual landmarks (such as the 5-choice wall itself) in the delay phase (during which the box is dark in the *dark* protocol) to maintain the information about the correct choice.) Additionally, the SD in SP and CP was 30 s in stage 1 and 20 s in stage 2 and at the beginning of stage 3 (see below).

There were further **sub-stages** within the main stages 1 and 2 - which were defined by the *amount of reward* given in the SP - and in main stage 3 - which were defined by the *stimulus duration* in the SP (SP-SD). Starting from a SP reward of 20 µl in stage 1 and 10 µl in stage 2, the amount was reduced at 10 µl and then 0 (stage 1) or to 0 (stage 2), if the respective criterion was met. (Note that the current sub-stage of stage 1, from which the animal departed, determined the sub-stage of stage 2 with which it continued, i.e. animals would receive 10 µl if they had received 20 or 10 µl in stage 1 before, but they would receive 0 µl, if they had arrived at 0 µl already in their prior training in stage 1.) This measure was done to avoid animals to adapt a strategy of obtaining their reward mainly from performing well in the SPs, while accepting a random (50 %) success rate for the CP; the same measure was used in the rat CAM-task (Chudasama et al., 2004, 2005; Chudasama and Robbins, 2004). In stage 3 (SP reward = 0 µl by default), the sub-stages involved a shortening of the SP-SD to 8 s and (in the first cohort, n = 2*12) to 4 s. This has the advantage of better standardizing the allowed amount of time to encode what is the correct stimulus location and provides a baseline (with 4 s SD), from which sustained attention could be challenged by reducing the SP-SD further on individual days, if desired, as done in the standard mouse 5-CSRTT (Bygrave et al., 2016; Grimm et al., 2018).

All **shifts between stages** required that the respective *performance criterion* was met in 3 consecutive training sessions. The criterion to transition between main stages was always based on working memory performance, requiring a CP accuracy_lit_ ≥ 70 % (analogous to the rat CAM-task) and a number of correct choice trials of ≥10. The criterion to transition between sub-stages was always based on the SP, requiring ≥25 correct SP responses and – for transitions within stage 3 (shortening of SD) a SP accuracy of ≥80 %.

Note that the rat CAM-task uses shorter SDs in both the SP (< 1 s) and the CP (3 s) (Chudasama et al., 2005), but – just like in the 5-CSRTT – mice have considerable difficulty with such short SDs, leading to high omission rates and hence slow learning progress. In the second cohort, animals were only trained up to the sub-stage with an SD of 8 s (Figure 3), because mice (in both cohorts) progressed only slowly towards sub-stages with shorter SDs. The major difference to the rat CAM task is that the whole task, consisting of both the SP and the CP, is trained right from the beginning, while the CAM-task started with training the SP only – i.e., the standard 5-CSRTT –until rats could perform with a SP-SD below 1 s, before adding the CP, resulting in a total training time of 6-8 mo (Chudasama et al., 2004, 2005; Chudasama and Robbins, 2004). Given our focus on the WM-component and difficulty of mice to achieve acceptably low omission rates with short SDs in the 5-CSRTT, we relaxed the requirement for a short SD and avoided potential re-learning problems upon altering the task-procedure (adding the CP) by training the full protocol from the beginning. The fact that at least some animals could learn the full working memory task (successful transitioning to stage 3, 20 s SD) within just a few weeks (Figure 3), suggests that this strategy is indeed successful, although it has to be acknowledged that we have no empirical evidence to confirm the superiority of this strategy over the two-phase version.

Finally it needs to be noted, that one half (n = 12) of our first cohort, deviated from the schedule above, as it was trained in the DNMTP paradigm first, which was terminated after 21 sessions due to lack of progress, and then entered the training schedule again from the beginning, albeit in the *dark* protocol (i.e. the house-light was switched off in the default training state and only illuminated for time-out (punishment) periods) and with the DMTP paradigm. The other half of that cohort was trained in the DMTP-paradigm in the *light* protocol throughout (i.e. the house-light was switched on in the default training state and only switched off for time-out (punishment) periods).

### Challenges in the 5-CSWM

For working memory it is common to see effects of pharmacological (Béracochéa et al., 2001; Zhang et al., 2013) or other manipulations (Kellendonk et al., 2009; Wolff et al., 2003) only with longer delays, which suggests that there needs to be a certain demand for holding specific information actively in memory for some longer time in order to necessitate high functioning of this cognitive process. Therefore, the first type of challenges used were the ones with an increased delay (*delay-challenges*). These challenges differ from the last training stage used (baseline stage), only in the length of the delay, adding either 10 or 20 s to the 2 s delay of the baseline protocol. Note that the effective delay is somewhat longer in each case, because the time that the animal takes to return to the reward receptacle and poke into it (which triggers the delay start) is not taken into account. As a third challenge the distance between the two illuminated holes in the choice phase was further decreased to zero (proximity challenge). This means that the two illuminated holes were directly next to each other. All other parameters were the same as in the baseline stage protocol in each case. Furthermore, during drug applications (see below), in addition to increasing the delay, we also shortened the SP-SD to 2 s to further standardize the permitted time to encode the future correct hole and to mildly challenge sustained attention, in order to evaluate potential modulation of this cognitive function simultaneously.

### Pharmacological experiments in the 5-CSWM task

Following the initial motivation for establishing this task as one that has predictive validity with respect to human or primate spatial working memory tests, the responses to different drugs, some of which were shown to influence working memory in the latter species at least under some conditions were tested.

Modafinil, the mGluR5-positive allosteric modulator (PAM) LSN 2463359 and the α2A-adrenoreceptor agonist guanfacine were tested in a within-subject design. All drugs were injected according to a latin-square scheme determining dose-assignment on a particular day, counterbalanced within the subgroups trained in the *light*- and *dark*-protocols by intraperitoneal (i.p.) route at a volume of 10 μl/g mouse (1 % of the bodyweight) and the stated solutions used for dissolving each drug were used as respective vehicle. Modafinil (Sigma, G) was dissolved in 20-24 % DMSO/saline and injected at the doses 0 (vehicle), 15, and 30 mg/kg 30 min before testing started, according to (González et al., 2014; Morgan et al., 2007). (A small subset of mice also received a further injection of 60 mg/kg, following (Béracochéa et al., 2001; Murphy et al., 2015; Piérard et al., 2007), but the result was not different from the 30 mg/kg performance, not shown). LSN 2463359 (Tocris, UK) was dissolved in 10 % DMSO / 0.3 % TWEEN80 / saline and administered in doses of 0 (vehicle), 0.33, 1.11, 3.33 and 10 mg/kg 60 min before testing started (based on doses used in (Gastambide et al., 2013; Gilmour et al., 2013). Guanfacine (Tocris, UK) was diluted in saline and administered at doses of 0 (vehicle), 0.33 and 1 mg/kg 60 min before testing started, following dose-response studies in the T-maze (Franowicz et al., 2002) and the 5-CSRTT (Pillidge et al., 2014). Working memory was challenged by applying an additional delay of 20 s (modafinil, LSN 2463359) or 10 s (guanfacine), and attention was mildly challenged by a sample-phase SD of 2 s. Running through the latin-square scheme, wash-out-days were incorporated after days of drug application (i.e. before the next injection) as follows: 1-2 d for modafinil and LSN 2463369, and 6-7 d for guanfacine.

### Statistical analysis

All data was analyzed in SPSS (IBM, US). Repeated-measures ANOVA across all groups (DMTP vs. DNMTP, Figure 1; *dark* vs. *light* protocol, Figure 2) and conditions (session blocks, Figure 1; challenges or drug doses, Figure 2) present in a given experiment were used throughout as initial analysis (see Supplementary Table 1 for all results). Simple-main-effects were used as primary post-hoc tests, if applicable (results indicated in figures only). For non-pharmacological experiments, paired t-tests were also calculated within groups for descriptive purposes (indicated in Supplementary Table 1 only).

# Supplementary figures


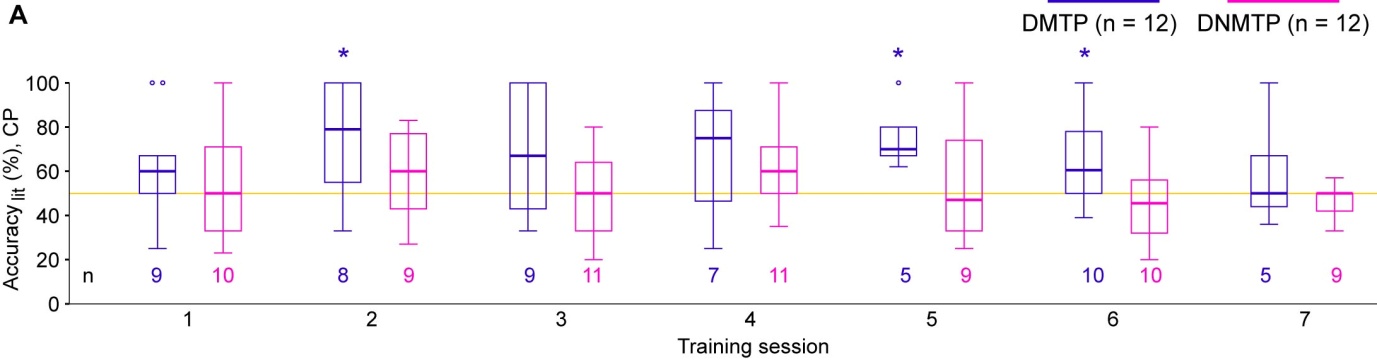


**Supplementary Figure 1. SWM performance during initial training sessions.** (**A**) Boxplots showing the accuracy_lit_ during the first 7 training sessions (same data as shown averaged in Figure 1E), as indicated, for mice trained in the DMTP (purple) and DNMTP (pink) paradigm; the number below each boxplot represents the number of animals that contributed to it, which excludes the mice that did not have a single correct response in the respective session (from 12 mice tested per group). The DMTP group showed a performance that was significantly higher than chance level (yellow line) on the days indicated (*; 0.01 < *p* < 0.05; one-sample t-test against 0.5). Note, however, the high variability in the DMTP group within and across training sessions, showing a lack of consistent performance.


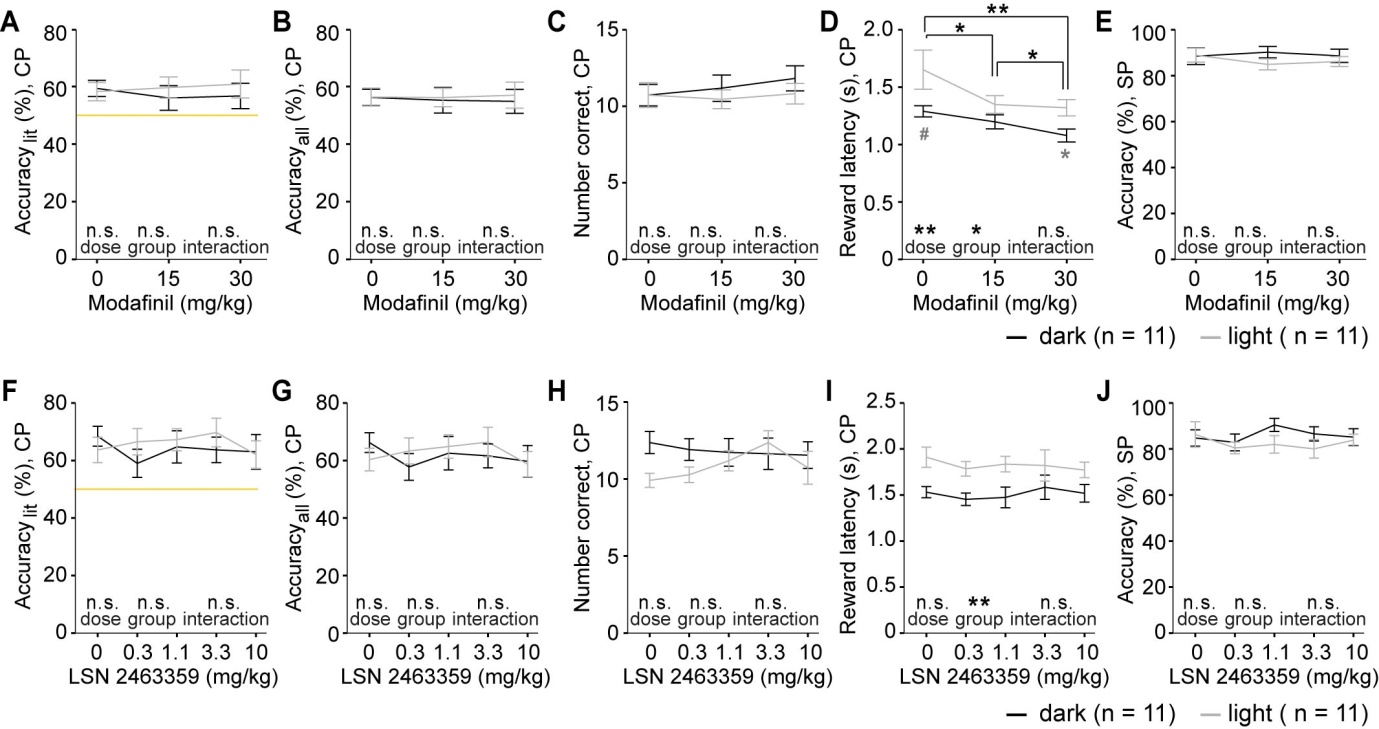


**Supplementary Figure 2. SWM performance after modafinil or LSN 2463359 application.**

Animals were pre-treated by modafinil (**A-E**, 30 min before testing) or the mGluR5-PAM LSN 2463359 (**F-J**, 60 min before testing) at the indicated doses. Delay-length was chosen at 22 s to challenge WM, and the SP stimulus duration (SD) was 2 s throughout to standardize encoding time and challenge sustained attention. For clarity, statistical effects of drug-dose, group and interactions found in repeated-measures ANOVAs are indicated above the respective words in each panel, while simple main-effects post-hoc results of pairwise dose-comparisons are indicated on horizontal lines (black) and effects of group at individual doses are indicated below the datapoints (grey). n.s. or no indication *p* > 0.1, # *p* < 0.1, *, *p* < 0.05; **, *p* < 0.01. All data is shown as mean±SEM. Modafinil (A-B) and LSN 2463359 (F-G) had no effect on SWM at the tested doses, but also not on SP accuracy (E, J). Nevertheless, general efficacy of modafinil could be confirmed by a significant decrease of the reward latency (D), consistent with increased motivational locomotor drive described at similar doses in mice before (Young et al., 2011). All data is shown as mean±SEM.


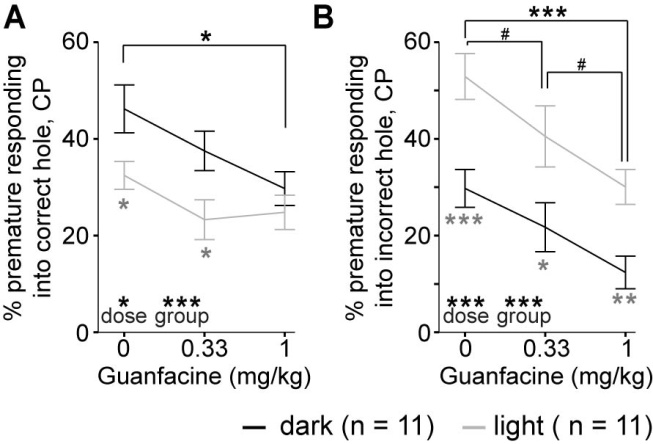


**Supplementary Figure 3. Premature responding in the choice phase after guanfacine.**

The average relative amount of choice phase trials in which mice made a correct (**A**) or incorrect (**B**) premature response, normalized to the number of started choice phase trials (i.e. number of correct sample phase responses) is displayed in %. In an overall repeated-measures ANOVA containing guanfacine dose, type of premature response and protocol (dark vs. light) as independent variables there were significant effects of dose (F = 17.3, *p* < 0.0005) and a response-type * protocol interaction (F = 48.3, *p* < 0.0005), while none of the other factors and interactions reached significance (*p* > 0.1). The interaction was driven by the fact, that animals in the *dark* protocol made significantly more *correct* premature responses than animals from the *light* protocol, while the reverse was true for *incorrect* premature responses. The results of the repeated-measures ANOVAs within each type of premature response are displayed at the bottom of each panel (dose-group interactions did not reach significance in either case, *p* > 0.1), the comparisons between individual dose-levels are indicated on top of each panel, and significant differences between protocols at each individual dose are indicated by grey asterisks (Simple-Main effects post-hoc comparison). n.s. or no indication *p* > 0.1, # *p* < 0.1, *, *p* < 0.05; **, *p* < 0.01, ***, *p* < 0.001. All data is shown as mean±SEM.

# Supplementary tables


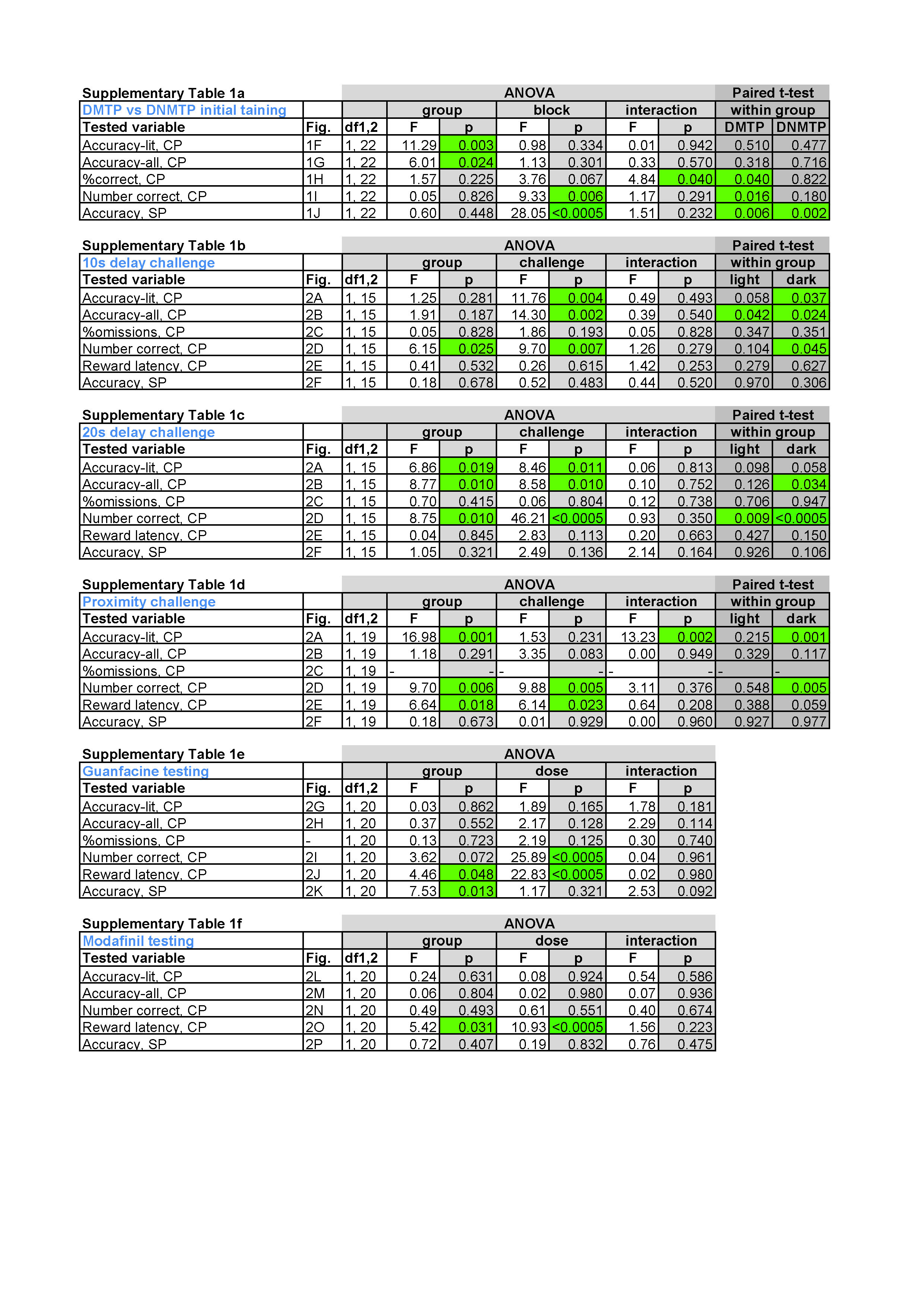


**Supplementary Table 1. Repeated-measures ANOVAs for behavioural tests.** The tested variables and the figure panel that displays the data (“Fig.”) are indicated in the two left columns; the experiment is identified in blue font. All data is taken from the primary male wildtype cohort (n = 2*12) but not all animals contributed to each experiment as described in the main text, hence degrees of freedom (df1, 2) differed. “F” and “p” columns render F- and *p*-values, respectively of the repeated-measures ANOVA, whereby significant *p*-values (< 0.05) are highlighted green. “Group” indicates the between-subject factor (SWM paradigm DMTP vs. DNMTP or house-light state: *light* vs. *dark* protocol). Block of training (first 7 vs. third 7 sessions), challenge-condition or drug-dose are within-subject factors, as indicated, and interactions are determined between both factors. For non-pharmacological tests, paired t-tests were calculated within the two groups separately, assessing significance for the respective within-subject factor; the resulting *p*-values are stated. SP, sample phase; CP, choice phase. Statistics for LSN 2463359 is not shown, since there were no significant or trend-level effects of dose or dose*group interactions (see Supplementary Figure 2).

| Stage | Experimental step | Important |
| --- | --- | --- |
| Pre-habituation | - Handling of mice (≥ 3 d) - Measure free-fed weights (FFW) at same time of day (3 d) - Remove regular food on 3^rd^ day of weighing (evening) - Provide strawberry milk reward in home cage (weighing boat) on last days of weighing until animals drink within ca. 60 min | - Animals need be calm at interaction with experimenter and consume milk reward in their home cage |
| Box-habituation | - Assign each animal to one operant box (for all stages) - Insert mice into operant box for 10 min with milk reward provided in illuminated receptacle; refill receptacle if mice finished the reward – repeat 2-3x/d until animals fully consume the reward in the box - Simultaneously, animal weights should gradually approach 85-90 % of FFW; weigh mice daily at same time from now on; extra-feed individual mice in separate cages 1-3 h per day before daily feeding of complete cage if necessary due to excessive weight drops/divergence | - Animals need to consume milk reward (ca. 100-200 μl per session) inside the operant box - Limit weight divergence – and esp. weight drops below 85 % FFW – by extra-feeding lighter mice 1-3 h after training |
| Operant habituation training | - 1 daily 30 min session of operant training: all 5 holes are illuminated until mice poke one of them, for which they obtain a 20 μl milk reward in then-illuminated receptacle; consumption of the reward restarts a new cycle without delay - Mice are trained until they obtain 40 rewards (trials) per session for 3 consecutive days - Mice can be trained a few (!) sessions further than criterion to allow moving multiple mice to the next stage *en-bloc* | - Mice should not be over-trained on this paradigm, as they have to unlearn it later (in our experience mice tend to rather associate *poking holes* with reward, not *illuminated* holes specifically!) - If necessary rather relax criterion to 30 trials & 2 consecutive days |
| 5-CSWM  Stage 1 | - Train mice in 5-CSWM cycle (see Methods) with 20 s SD in SP and CP, 2 s delay and 15 s ITI; reward sizes are 20 μl (SP, initially) and 60 μl; omissions and incorrect responses in each phase are punished by omission of reward and immediate switch of houselight state for 5 s after which a new ITI and trial starts - We recommend training in the DMTP paradigm and the *dark* protocol (houselight off by default, only on for time-outs) - SP-reward size is decreased to 10 μl and (*optionally*) 0 μl if mice have obtained ≥25 #correct in the SP on 3 consecutive days - Mice need to achieve ≥10 #correct and ≥70 % accuracy_lit_ on 3 consecutive days to transition to Stage 2 | - Weigh & train animals at the same time of day - The ITI can also be shortened to 10 s after CP and 5 s after time-out in SP (throughout stages) - The key goal at this stage is for the animals to obtain *many* trials & #correct to facilitate training progress |
| 5-CSWM  Stage 2 | - Training as stage 1, but with more CP configurations and SP-reward of 10 μl or (*optionally*) 0 μl (see Methods) - Mice need to achieve ≥10 #correct and ≥70 % accuracy_lit_ on 3 consecutive days to transition to Stage 3 | - If animals take many sessions (>100) to reach criterion, this stage can also be used as final |
| 5-CSWM  Stage 3 | - Training as stage 2, but with more CP configurations and SP-reward of 0 μl (see Methods) - SP-SD is reduced to 8 s if SP-accuracy is ≥80 % - Mice need to achieve ≥10 #correct and ≥70 % accuracy_lit_ on 3 consecutive days to transition to challenges   *Optional:*   - The number of CP-configurations might be increased further by also adding those with neighbouring holes (e.g. holes 3 & 4) - SP-SD and CP-SD can be reduced further (e.g. to 4 s) without affecting accuracies much – check response latencies to see how much time animals actually need! | - a SP-reward of 10 μl might also be used in Stages 2&3 and further, e.g. to enforce/motivate receptacle entries at the *end* of the delay phase when extending the delay in challenge protocols - reduction of SDs helps to standardize encoding and decision times, eliminating trials where mice don’t participate in the task; also allows to test attention by reducing SP-SD to 1 s as a challenge later |
| Challenge protocols | - run challenge protocols on 1 or 2 consecutive days per challenge with training in baseline protocol (Stage 3) on ≥2 d in between different challenges - *Delay challenge*: increase delay to 5 s, 10 s and/or 20 s before or after required receptacle entry   *Theoretical further options:*   - *Distraction*: briefly switch the houselight (0.5-1.0 s) 1-2x at random times or present a tone during delay - *Attention challenge (analogous to 5-CSRTT)*: reduce SP-SD to 1 s (from a baseline of 4 or 2 s) - Use challenges esp. when conducting potentially WM-enhancing manipulations (e.g. pharmacology) | - Delay challenges are key, but animals may adapt to it upon multiple replications - Adding more CP-configurations (pairs of holes) & proximity do not have a strong challenging effect after Stage 3 - For distraction, monitor orienting response as older mice might be deaf; don’t use illumination of all 5 holes as distraction, as it is mistaken as GO-signal (i.e. a CP) |

**Supplementary Table 2. Training schedule and checklist for setting up the 5-CSWM task.** The training stages are listed in the order of their succession during the training of a cohort, with the main parameters and instructions for their conduct (middle column) and important points to be aware off (right column). Note that multiple other challenges might be tried, e.g. using more than 2 illuminated holes as CP-configurations (might need to be explicitly trained though), combinations of the challenges, or variable delays within one session. For data acquisition, ensure that for the *sample phase* number of total, correct, incorrect and omitted trials, reward and response latencies (the latter separately for incorrect and correct trials), and for the *choice phase* number of total, correct, incorrect (separately for response to illuminated and non-illuminated holes) and omitted trials, reward and response latencies (separately for incorrect and correct trials), and premature responses (separately for incorrect and correct CPs) are recorded. *Abbreviations:* CP, choice phase; FFW, free-feeding weight (baseline weight established before food deprivation); ITI, inter-trial interval; SD, stimulus duration; SP, sample phase.

# Supplementary References

Béracochéa, D., Cagnard, B., Célérier, A., le Merrer, J., Pérès, M., and Piérard, C. (2001). First evidence of a delay-dependent working memory-enhancing effect of modafinil in mice. *Neuroreport* 12, 375–8.

Bygrave, A. M., Masiulis, S., Nicholson, E., Berkemann, M., Sprengel, R., Harrison, P., et al. (2016). Knockout of NMDA-receptors from parvalbumin interneurons sensitizes to schizophrenia-related deficits induced by MK-801. *Transl. Psychiatry* 6, e778.

Chudasama, Y., Dalley, J. W., Nathwani, F., Bouger, P., and Robbins, T. W. (2004). Cholinergic modulation of visual attention and working memory: dissociable effects of basal forebrain 192 IgG-saporin, lesions and intraprefrontal infusion of scopolamine. *Learn. Mem.* 11, 78–86. doi:10.1101/lm.70904.such.

Chudasama, Y., Nathwani, F., and Robbins, T. W. (2005). d-Amphetamine remediates attentional performance in rats with dorsal prefrontal lesions. *Behav. Brain Res.* 158, 97–107.

Chudasama, Y., and Robbins, T. W. (2004). Dopaminergic Modulation of Visual Attention and Working Memory in the Rodent Prefrontal Cortex. *Neuropsychopharmacology* 29, 1628–1636. doi:10.1038/sj.npp.1300490.

Franowicz, J. S., Kessler, L. E., Borja, C. M. D., Kobilka, B. K., Limbird, L. E., and Arnsten, A. F. T. (2002). Mutation of the α2A-Adrenoceptor Impairs Working Memory Performance and Annuls Cognitive Enhancement by Guanfacine. *J. Neurosci.* 22, 8771–8777.

Gastambide, F., Gilmour, G., Robbins, T. W., and Tricklebank, M. D. (2013). The mGlu5 positive allosteric modulator LSN2463359 differentially modulates motor, instrumental and cognitive effects of NMDA receptor antagonists in the rat. *Neuropharmacology* 64, 240–247. doi:10.1016/j.neuropharm.2012.07.039.

Gilmour, G., Broad, L. M., Wafford, K. A., Britton, T., Colvin, E. M., Fivush, A., et al. (2013). In vitro characterisation of the novel positive allosteric modulators of the mGlu5 receptor, LSN2463359 and LSN2814617, and their effects on sleep architecture and operant responding in the rat. *Neuropharmacology* 64, 224–239. doi:10.1016/j.neuropharm.2012.07.030.

González, B., Raineri, M., Cadet, J. L., García-Rill, E., Urbano, F. J., and Bisagno, V. (2014). Modafinil improves methamphetamine-induced object recognition deficits and restores prefrontal cortex ERK signaling in mice. *Neuropharmacology*. doi:10.1016/j.neuropharm.2014.02.002.

Grimm, C. M., Aksamaz, S., Schulz, S., Teutsch, J., Sicinski, P., Liss, B., et al. (2018). Schizophrenia-related cognitive dysfunction in the Cyclin-D2 knockout mouse model of ventral hippocampal hyperactivity. *Transl. Psychiatry* 8, 212. doi:10.1038/s41398-018-0268-6.

Kellendonk, C., Simpson, E. H., and Kandel, E. R. (2009). Modeling cognitive endophenotypes of schizophrenia in mice. *Trends Neurosci.* 32, 347–58. doi:10.1016/j.tins.2009.02.003.

Morgan, R. E., Crowley, J. M., Smith, R. H., LaRoche, R. B., and Dopheide, M. M. (2007). Modafinil improves attention, inhibitory control, and reaction time in healthy, middle-aged rats. *Pharmacol. Biochem. Behav.* 86, 531–541. doi:10.1016/j.pbb.2007.01.015.

Murphy, H. M., Ekstrand, D., Tarchick, M., and Wideman, C. H. (2015). Modafinil as a cognitive enhancer of spatial working memory in rats. *Physiol. Behav.* 142, 126–130. doi:10.1016/j.physbeh.2015.02.003.

Piérard, C., Liscia, P., Philippin, J.-N., Mons, N., Lafon, T., Chauveau, F., et al. (2007). Modafinil restores memory performance and neural activity impaired by sleep deprivation in mice. *Pharmacol. Biochem. Behav.* 88, 55–63. doi:10.1016/j.pbb.2007.07.006.

Pillidge, K., Porter, A. J., Dudley, J. A., Tsai, Y.-C., Heal, D. J., and Stanford, S. C. (2014). The behavioural response of mice lacking NK1 receptors to guanfacine resembles its clinical profile in treatment of ADHD. *Br. J. Pharmacol.* 171, 4785–4796. doi:10.1111/bph.12860.

Wolff, M., Benhassine, N., Costet, P., Hen, R., Segu, L., and Buhot, M.-C. (2003). Delay-Dependent Working Memory Impairment in Young-Adult and Aged 5-HT1BKO Mice as Assessed in a Radial-Arm Water Maze. *Learn. Mem.* 10, 401–409. doi:10.1101/lm.60103.

Young, J. W., Kooistra, K., and Geyer, M. A. (2011). Dopamine Receptor Mediation of the Exploratory/Hyperactivity Effects of Modafinil. *Neuropsychopharmacology* 36, 1385–1396. doi:10.1038/npp.2011.23.

Zhang, X.-H., Liu, S.-S., Yi, F., Zhuo, M., and Li, B.-M. (2013). Delay-dependent impairment of spatial working memory with inhibition of NR2B-containing NMDA receptors in hippocampal CA1 region of rats. *Mol. Brain* 6, 13. doi:10.1186/1756-6606-6-13.
